# Understanding Microbial Divisions of Labor

**DOI:** 10.3389/fmicb.2016.02070

**Published:** 2016-12-21

**Authors:** Zheren Zhang, Dennis Claessen, Daniel E. Rozen

**Affiliations:** Institute of Biology, Leiden UniversityLeiden, Netherlands

**Keywords:** division of labor, microbial multicellularity, kin selection, *Streptomyces*, microbial social evolution

## Abstract

Divisions of labor are ubiquitous in nature and can be found at nearly every level of biological organization, from the individuals of a shared society to the cells of a single multicellular organism. Many different types of microbes have also evolved a division of labor among its colony members. Here we review several examples of microbial divisions of labor, including cases from both multicellular and unicellular microbes. We first discuss evolutionary arguments, derived from kin selection, that allow divisions of labor to be maintained in the face of non-cooperative cheater cells. Next we examine the widespread natural variation within species in their expression of divisions of labor and compare this to the idea of optimal caste ratios in social insects. We highlight gaps in our understanding of microbial caste ratios and argue for a shift in emphasis from understanding the maintenance of divisions of labor, generally, to instead focusing on its specific ecological benefits for microbial genotypes and colonies. Thus, in addition to the canonical divisions of labor between, e.g., reproductive and vegetative tasks, we may also anticipate divisions of labor to evolve to reduce the costly production of secondary metabolites or secreted enzymes, ideas we consider in the context of streptomycetes. The study of microbial divisions of labor offers opportunities for new experimental and molecular insights across both well-studied and novel model systems.

## Introduction

It is often stated that there is “strength in numbers.” Bigger armies tend to conquer smaller ones, while giant flocks of starlings migrate more efficiently than a single bird on its own. But what is the source of the added value in having more individuals? In some cases it is pure power, e.g., a pride of lions that is more effective at subduing large prey through the simple benefit of added strength. However, in many other examples, the benefit of numbers derives from the fact that larger groups of individuals can segregate tasks, thereby allowing them to diversify into teams of coordinated specialists that can accomplish more together than the simple sum of their parts. The understanding of this “division of labor” has its origin in studies of human economics, but the central idea is of equal importance across the diversity of life and at virtually every scale of biological organization (Smith et al., 2008; van Gestel et al., 2015a; West and Cooper, 2016). Social insects clearly exemplify divisions of labor at the level of a society of individuals (Smith et al., 2008). Within a colony of leaf cutter ants, potentially containing millions of individuals, there are soldiers who defend the nest, foragers that travel far and wide gathering leaves, gardeners of many types to tend to the specialized fungal gardens these ants require for nourishment, and nurses to rear offspring, among many others (Wilson, 1980). Narrowing our view to the level of a single individual, multicellular organisms also are characterized by a division of labor among distinct cell and tissue types that each play specialized roles in maintaining the fitness of the whole organism. However, divisions of labor are not the unique privilege of animals. As we discuss below, many microbes also divide tasks among clone-mates, and these divisions can have far-reaching effects for colony-level fitness.

In the interest of space, we only briefly discuss the evolutionary advantages of a division of labor and instead refer interested readers to the numerous excellent reviews on the topic in multicellular organisms or microbes (Smith et al., 2008; van Gestel et al., 2015a; West and Cooper, 2016). However, we review several key features in order to guide the remaining text. Divisions of labor in a society or a single organism require the coexistence of multiple types, or subpopulations, that interact and are specialized to carry out complementary tasks (van Gestel et al., 2015a). In microbial colonies, each sub-type can be derived from a single parental cell in response to environmental change (e.g., starvation) via deterministic or stochastic processes (Veening et al., 2008). Differentiation into functionally distinct cell types, whether it arises from genetic or regulatory changes, requires cooperation among these types, which is maintained by their shared evolutionary interests via kin selection, whereby individuals sacrifice individual fitness for the sake of related individuals (West and Cooper, 2016). Importantly, differentiated colonies have higher fitness than those lacking a division of labor. This benefit typically results from the added efficiency of dividing tasks between cells rather than a single cell either switching between these tasks or carrying them out simultaneously, although other advantages can be envisioned (Velicer and Vos, 2009; Rossetti and Bagheri, 2012; van Gestel et al., 2015a; Kim et al., 2016; West and Cooper, 2016). In addition, it is important to note that phenotypic diversification does not necessarily represent a division of labor, as heterogeneity among cells can provide individual benefits that may have no effects on the group or even reduce colony fitness. Accordingly, by our strict definition, divided complementary tasks must increase population fitness to be identified as a division of labor. As made clear in **Supplementary Table 1**, these criteria have only been experimentally verified in a handful of cases, although divisions of labor are nevertheless often assumed to be present. In examples we discuss below, e.g., the division of labor between vegetative growth and sporulation, differentiated tasks are mutually incompatible and cannot be carried out by a single cell at once. Using these generalized features, we next consider specific examples of divisions of labor before focusing our attention on streptomycetes.

## Divisions of Labor in Multicellular Microbes

Although microbes often live solitary lives, many phylogenetically divergent groups have independently evolved different levels of coordinated or patterned multicellularity (Claessen et al., 2014). These can be facultative, in the case of biofilms that develop from the aggregation and proliferation of independent cells, or can be an obligate component of the microbial life-cycle. The latter group, microbes that display obligate patterned multicellularity, offer the most dramatic examples of divisions of labor. This is because these groups are characterized by terminal differentiation into reproductive and non-reproductive cells that mimic the divisions between germ and soma in plants or animals (Shimkets, 1990; Strassmann et al., 2011; Claessen et al., 2014; Herrero et al., 2016). In addition to offering insights into the evolution of microbial multicellularity and a division of labor, the examples we consider also provide the best evidence of the central factors that ensure that these divisions are stably maintained. Here, there is necessary overlap with conditions that maintain cooperative behaviors against “cheats” more generally (West et al., 2006, 2007; Oliveira et al., 2014; Borgeaud et al., 2015).

## Maintaining Divisions of Labor with Aggregative Multicellularity

Myxobacteria are social bacteria with a multicellular lifestyle (Velicer and Vos, 2009; Claessen et al., 2014). When growing in the presence of abundant resources, vegetative cells in the best studied species, *Myxococcus xanthus*, hunt socially via the coordinated secretion of lytic enzymes that digest bacterial and fungal prey (Velicer and Vos, 2009; Morgan et al., 2010; Xiao et al., 2011). Upon starvation, they undergo a dramatic transition where individual cells migrate together to create fruiting bodies containing ∼10^5^ cells. Fruiting bodies in *M. xanthus* contain three differentiated cell types: spores, which comprise around 10% of the fruiting body, peripheral rods that comprise another 10–30%, and then the rest that die and lyse during development via a process assumed to be programmed cell death (PCD) (Shimkets, 1990; Claessen et al., 2014; Muñoz-Dorado et al., 2016).

Spores are the most easily understood of the myxobacterial cell types, as these are the cells that persist through environmental deprivation and stress. Moreover, the benefit of their survival is direct and immediate. By contrast, any benefits of coordinated development for the other cell types are likely to be indirect, especially for the 60–80% of cells fated to die. If the death of these cells is caused by PCD, what explains their altruistic self-sacrifice? The simplest explanation is kin selection: as stalk cells are the clone mates of spores, their sacrifice is repaid indirectly when related spores survive (Velicer and Vos, 2009). Accordingly, it is assumed that PCD in these stalk cells directly increases spore numbers or the probability of spore survival, although the mechanisms by which this might occur remain unclear (Lewis, 2000). One possibility is that stalk cells aid in spore dispersal, perhaps by elevating them above the substrate. Another argument is that material from lysed cells, e.g., lipid bodies, is incorporated into the spore or spore coat which works to increase spore hardiness (Bhat et al., 2014a,b). Despite suggestive evidence for both possibilities, neither option has been validated experimentally.

It could be argued that a detailed understanding of the mechanisms by which stalk or peripheral rod cells contribute to spore survival is not needed, as we can already adequately explain the evolutionary maintenance of a division of labor among clonal groups of cells by kin selection. However, two areas of research would benefit from a fuller understanding of these mechanisms: (1) the division of labor in non-clonal groups, an area that has already been studied extensively, and (2) explaining the relative frequencies of cell types within and across genotypes, a topic that has been largely neglected. Why, for example, do 10% of cells become spores instead of 5%, 50% or even 100%? Is this value fixed across strains or do strains vary in their allocation to spores or stalk cells? And can cell type frequencies for any given strain respond adaptively to environmental contingencies? Analogous questions have long been posed in the context of social insects using the terminology of caste ratios (Passera et al., 1996; Strand et al., 2000). We believe a similar framework would be valuable for microbes. We address each of these areas in turn.

## Labile Divisions of Labor

Altruism among clone-mates can be explained by kin selection. However, both in the lab and in nature, there is evidence that myxobacterial fruiting bodies can be comprised of mixed genotypes where the benefits of altruistic behaviors are strongly reduced (Velicer et al., 2000; Velicer and Vos, 2009; Kraemer and Velicer, 2011). Where relatedness among strains in fruiting bodies is low, there is strong selection for the evolution of “cheats” that seek to benefit at the expense of others by increasing their own representation within the population of spores (Fiegna and Velicer, 2005; Vos and Velicer, 2009; Kraemer and Velicer, 2011). In one study, socially defective mutants that arose during selection for rapid growth lost the ability to sporulate in isolation; however, when these cells were mixed with wild-type clones they were able to increase in frequency, potentially by utilizing the developmental signals of wild-type cells, although the mechanisms are not fully understood (Velicer et al., 2000, 2002; Kraemer et al., 2014). Natural isolates also show significant variation in spore output across several orders of magnitude, and as with laboratory evolved clones, these wild-type variants can also exploit one another in chimeric fruiting bodies (Velicer and Vos, 2009; Kraemer et al., 2010; Kraemer and Velicer, 2011). These cheats have led to the evolution of diverse mechanisms to distinguish kin from non-kin (Rendueles et al., 2015; Wall, 2016), an issue we will consider later on. Interestingly, these interactions also provide suggestive evidence that the division of labor in myxobacteria is socially contingent; whereas strains in isolation produce a fixed number of spores, this value can vary during competitive interactions. However, at present, it remains unclear how competitive interactions affect the allocation behavior of different genotypes to different cell types, the caste ratio, and if this is dependent on the identity of competing strains. For example, it is possible that exploitative strains grown as chimeras increase their individual spore output by decreasing allocation to peripheral cells or cells that die via PCD; in other words, competition leads to an adaptive change in the caste ratio. Alternatively, the caste ratio of these strains may remain unchanged, even while total spore number increases, if these strains are able to increase total cell numbers at the expense of their competitors. The key issue with respect to divisions of labor is to distinguish how cells behave in isolation from their behavior in mixtures. Does the caste ratio change, and if so, does it change in both competitors or in one competitor at the expense of the other? Additionally, it is crucial to quantify how these changes influence spore survival—the presumed reason these cells divide labor at all.

Some of these questions have been considered in an analogous microbial system: the social eukaryote *Dictyostelium discoideum*. Like myxobacteria, Dictyostelids live as asocial bacterivores that, upon starvation, aggregate together and differentiate into a multicellular fruiting body containing spores and altruistic stalk cells (plus several other minority cell types) (Li and Purugganan, 2011; Strassmann and Queller, 2011). When genetically different strains are mixed together to form chimeric fruiting bodies, one strain often appears to gain unfair representation in the spores (Strassmann et al., 2000; Fortunato et al., 2003; Buttery et al., 2009). While these “winner” strains have been labeled cheaters, alternative explanations, not based on exploitation, could lead to the same outcome. Like myxobacteria, there is extensive natural variation among *Dictyostelium* genotypes for caste ratio (Buttery et al., 2009). Cells of some strains primarily differentiate into spores during development, while in other strains, most cells in fruiting bodies differentiate into stalk cells. When strains of these two extremes are mixed, it is easy to see that the former would produce the majority of spores. However, this may not be the result of changes to caste ratios, as maintaining a “fixed” strategy—behaving in mixtures just as you would when alone—also leads to competitive differences between strains. Indeed, knowing the caste ratio of a strain grown in isolation is almost perfectly predictive of its spore production in chimeric fruiting bodies (Buttery et al., 2009). This predictability makes clear, in a way that has not yet been possible in myxobacteria, that deviations in divisions of labor can have dramatic consequences for microbial social behaviors and interactions.

If *Dictyostelium* strains that differentiate a greater fraction of cells into spores are apparently socially dominant, why don’t all strains utilize a similar division of labor? The answer, it turns out, lies in the fact that allocation decisions are coupled to trade-offs in spore size, number and viability. Strains with high proportions of spore:stalk, those that “win” during social competition, tend to make many smaller spores that individually have reduced viability (Wolf et al., 2015). By contrast, strains that divide labor by differentiating a greater fraction of cells into stalk, tend to make fewer larger spores that each have higher viability. Accordingly, what “winners” gain in terms of spore numbers, they lose in terms of spore viability, and this leads to an overall equivalence in the fitness of strains. Of course, this equivalence leads back to the original question of why different strategies exist—and here there are no clear answers, because we simply lack an understanding of why these microbes divide labor to begin with. Recent work in *Dictyostelium* focusing on a third type of cell that remains vegetative and fails to aggregate during starvation, have suggested that variance in starvation times (i.e., seasonality) can allow for the coexistence of different division of labor strategies (Tarnita et al., 2015; Martinez-Garcia and Tarnita, 2016). By this mechanism one could envision that if spores of different sizes also differ in the duration of dormancy or their sensitivity to cues required to exit dormancy, then different divisions of labor could arise across a heterogeneous landscape. At present, this remains untested. It does, however, emphasize the need to supplement kin selection arguments for the evolutionary maintenance of microbial divisions of labor with a more detailed understanding of the ecological factors that lead to the coexistence of different division of labor strategies in nature.

## Cheating and Kin Recognition

Whether via fixed strategies, as outlined above, or via “facultative” adjustments to caste ratios (Buttery et al., 2009), it is clear that microbial divisions of labor can have profound effects on social interactions between strains. In response to this, many microbes have evolved mechanisms of kin discrimination to ensure that altruistic behaviors are preferentially directed toward clone mates (Strassmann et al., 2011; Strassmann, 2016; Wall, 2016). In multicellular microbes like myxobacteria or *Dictyostelium*, both active and passive mechanisms (Buttery et al., 2012; Smith et al., 2016) work to keep different genotypes apart. Highly polymorphic cell-surface-mediated matching systems in both microbes allow strains to distinguish self from non-self. While some of the genes underlying these responses are known (e.g., *tra* or *tgr* loci in myxobacteria or *Dictyostelium*, respectively) (Ho et al., 2013; Strassmann, 2016; Wall, 2016) it is also apparent that mechanisms of exclusion can evolve rapidly via diverse mechanistic routes (Rendueles et al., 2015). In other cases, strains can remain spatially segregated by passive means if the migration of cells is highly restricted or if population sizes remain low, thus reducing the encounter rate of different strains (Buttery et al., 2012; Smith et al., 2016). Regardless of the mechanisms used by strains to insulate themselves from social exploitation, it is clear that these mechanisms are effective; fruiting bodies of both myxobacteria and *Dictyostelium* are most often clonal in nature (Gilbert et al., 2007, 2009; Kraemer and Velicer, 2011). Thus, it is likely that the different divisions of labor that distinguish strains are maintained for reasons that may have little to do with social challenges, but rather because of ecological benefits to specific strategies that are contingent on the environment where strains are growing.

## Divisions of Labor in Patterned Multicellularity

Several groups of multicellular microbes display forms of patterned multicellularity where divisions of labor arise following the outgrowth of a single cell, much like the multicellularity that characterizes animals or plants (Claessen et al., 2014). In contrast to the aggregative multicellular microbes discussed above, cells in these species are physically attached to one another and form filaments with semi-permeable cross-walls and growth at the filament tips. These features, which ensure clonality, largely insulates these groups from social exploitation from within. In addition, because of their high relatedness, microbial colonies with patterned multicellularity tend to contain more cells/biomass as well as a larger diversity of cell types (Fisher et al., 2013).

In filamentous cyanobacteria, photosynthetic microbes responsible for a large fraction of our planet’s primary production, some species have evolved strategies to differentiated into two cell types that segregate chemically incompatible tasks – photosynthesis and nitrogen fixation (Flores and Herrero, 2010; Rossetti and Bagheri, 2012). Some of them, e.g., *Anabaena* spp. terminally differentiate around 5–10% of their cells into specialized cells, called heterocysts, that carry out nitrogen fixation. As with myxobacterial cells that undergo PCD, heterocysts in *Anabaena* are unable to divide and are thus reproductively sterile (Rossetti et al., 2010). Alternatively, in some non-heterocystous species, e.g., *Plectonema boryanum*, a temporal division of labor is employed to allow cells to pursue both photosynthesis and nitrogen fixation by switching between both functions on the basis of an externally driven circadian rhythm. Mathematical studies have suggested that compared to temporal divisions of labor, spatially segregating incompatible tasks, such as in *Anabaena*, can overcome biochemical constrains between distinct pathways and thereby maximize the production of biomass from the available light or nitrogen (Rossetti and Bagheri, 2012). In addition, this physical division of labor offsets time or resource costs associated with alternating between two distinct metabolic systems, while theoretical studies have suggested that the ratio of heterocysts to vegetative cells has evolved to maximize carrying capacity under conditions of high light (Rossetti et al., 2010). Interestingly, cyanobacterial divisions of labor are labile and can be regulated depending on environmental conditions. For example, in the presence of a utilizable nitrogen source, thus reducing the requirement for endogenous nitrogen fixation, the filaments of *Anabaena* or *Nostoc* form homogenous filaments of vegetative cells that remain undifferentiated (Meeks and Elhai, 2002; Flores and Herrero, 2010). Such flexibility is analogous to the flexible caste ratios seen in social insects, e.g., in the ant *Pheidole pallidula*, where the production of soldier pupae and adult soldiers both increase under threat of foreign workers from unrelated colonies (Passera et al., 1996). Filamentous cyanobacteria generate several other classes of differentiated cell types, such as akinetes, that act as durable spores and arise during conditions of starvation (Adams and Duggan, 1999; Flores and Herrero, 2010). However, it is as yet unclear how the fraction of cells that adopt these states is determined.

Another example of a division of labor in patterned microbial multicellularity can be found in streptomycetes which are filamentous spore-forming bacteria that are widespread in terrestrial and aquatic environments (Claessen et al., 2014; Barka et al., 2016). Streptomycete colonies arise following the germination of a single spore which gives rise to a multi-chromosomal mycelium that superficially resembles that of filamentous fungi (Hopwood, 2007). These multicellular organisms forage on complex organic materials that are converted into small molecules using secreted proteases, cellulases, and chitinases (Chater et al., 2010). Upon nutrient depletion behind an actively growing colony front, a developmental program is initiated allowing these bacteria to escape harsh environmental conditions (Flärdh and Buttner, 2009; Barka et al., 2016). This leads to the formation of aerial hyphae that differentiate into unigenomic spores. The energetic burden associated with the formation of these reproductive structures is thought to be supported by the partial degradation of the vegetative mycelium via PCD (Manteca et al., 2006; Yagüe et al., 2012). Because streptomycete colonies are physically attached to one another and are the clonal products of division and growth from a single spore, kin selection can also explain this apparently altruistic PCD (Nedelcu et al., 2011). In addition, the architecture of streptomycete colonies appears to largely insulate strains from mutations that give rise to less PCD or from exploitation from strains via colony fusion (Braendle and Szybalski, 1957, 2006). PCD within streptomycete colonies is coupled to the production of numerous secondary metabolites, some of which have strong antimicrobial properties (Hopwood, 2007). As starvation is an environmental cue for sporulation, these antimicrobials are thought to prevent competitive soil bacteria in the same nutrient-deprived environment from benefitting from the nutrients released during PCD (Rigali et al., 2006, 2008). The central molecular mechanism that connects PCD to antibiotic production is the pleiotropic transcriptional repressor DasR, which prevents antibiotic production during vegetative growth. Colony dismantling during PCD leads to the extracellular accumulation of cell wall-derived *N*-Acetylglucosamine (GlcNAc) around the colony periphery. Internalization and modification of GlcNAc subsequently yields glucosamine-6-phosphate (GlcN-6P), which can allosterically bind to DasR thereby relieving its repressing activity (Tenconi et al., 2015). As such, this switch is considered a robust timing mechanism to maximize the colony-wide benefits while reducing the potential harm of PCD (Rigali et al., 2006, 2008).

The vegetative and reproductive growth phases in streptomycetes represent a clear example of a division of labor: the vegetative hyphae are programmed to forage, while the reproductive hyphae lead to stable spores that can persist through starvation and potentially migrate to more fruitful resource patches (Hopwood, 2007). Notably, these processes are also mutually incompatible due to their distinct physical positions in the colony itself. Unlike vegetative hyphae which grow radially from a colony center through the substrate, aerial hyphae are physically separated from potential nutritional resources. Instead, they emerge from the colony surface and protrude up into the air (Claessen et al., 2006). To achieve this, they become enveloped by a hydrophobic surface layer that possibly serves two roles: it maximizes successful spore dispersal, but also prevents hyphae from growing back into the substrate (Claessen et al., 2004). The vegetative hyphae, on the other hand, have a more hydrophilic nature which may make them better suited to thrive in soils, whose natural minerals, such as silica, are hydrophilic.

## Divisions of Labor Beyond Sporulation

In parallel with the myxobacteria or *Dictyostelium*, the most obvious divisions of labor in streptomycetes concern those arising during sporulation. However, both in this and other systems, additional divisions of labor are likely to arise if colony-wide benefits can be obtained at the expense of a small fraction of cells (van Gestel et al., 2015a; West and Cooper, 2016). In particular, we expect these to be found for secreted products, like antibiotics or enzymes, whose effects can be shared by producers and non-producers alike and whose production is metabolically costly. Thus, by differentiating a subset of specialized cells dedicated to production at the cost of their own replication, colonies can potentially increase overall efficiency. Antibiotic production in *Streptomyces* offers a clear test of this possibility. The species in this genus are prolific antibiotic producers that are responsible for some 70% of all antibiotics used in human and veterinary medicine (Barka et al., 2016). Because antibiotic biosynthesis is metabolically costly and can trade-off with growth, it is conceivable that production and secretion by only a fraction of the hyphae would offer resource savings, yet be sufficient to provide benefits to the entire colony. Concomitantly, the antibiotic non-producing hyphae could continue foraging while transporting nutrients to other parts of the colony. At present, there are few data to support the existence of a trade-off between growth and secretion (aside from unpublished results from our own labs) nor data on the form this trade-off takes (Michod, 2006), either for antibiotics or other secreted products; however, we suspect this is more for lack of looking than true absence. Indeed, in similarly structured filamentous networks of fungal hyphae, increasing evidence supports the idea that there is considerable heterogeneity across the colony in the production of costly secreted enzymes (Vinck et al., 2005, 2011; Levin et al., 2007; Bleichrodt et al., 2012). Gene expression and translational activity in *Aspergillus* is spatially variable leading to subclasses of differentiated hyphae that adopt distinct functional roles (Vinck et al., 2005). At the colony periphery, minority fractions of hyphae highly express secreted proteins, including glucoamylase, acid amylase, *a*-glucuronidase, and feruloyl esterase, that are essential for resource acquisition. This apparent division of labor enables a small fraction of the total colony biomass to focus on enzyme secretion and foraging, while the majority remains in a so-called “battery-saving” transcriptional status (Vinck et al., 2011; Bleichrodt et al., 2015). Convincing experiments have clarified that hyphal heterogeneity in *Aspergillus oryzae* is regulated by restrictions to cytoplasmic streaming, regulated by Woronin bodies that transiently block septa between fungal compartments (Bleichrodt et al., 2015). Although further work is required to quantify the potential ecological advantages of this division of labor, as well as the form of the trade-offs that may govern it (Michod, 2006), it has been argued that it facilitates colony-wide responses to unpredictable environmental stress (Wösten et al., 2013). Additionally, it remains unclear which environmental factors serve as cues for differentiation, and whether different strains vary in these responses to these cues. Despite the considerable differences between filamentous bacteria and fungi, their convergent morphologies suggest that similar divisions of labor may have evolved in both groups to reduce the costs and increase the efficiency of secreted products, especially given recent results conclusively demonstrating effective cytoplasmic streaming in streptomycetes (Celler et al., 2016; Yagüe et al., 2016).

As research into microbial divisions of labor expand, more examples are identified, even in species that lack the types of patterned multicellularity outlined above. For example, in *Bacillus subtilis* biofilms, flagellum-independent migration can be conducted by the collective action of two cell types: surfactin-producing cells and those that produce matrix (van Gestel et al., 2015b). While surfactin production works as a lubricant to reduce the obstruction between cells and substrate, matrix producing cells assemble themselves into so-called van Gogh bundles that can migrate over greater distances than would be possible without this division of labor. During this process, *srfA*, which coordinates surfactic production, is up-regulated via quorum sensing, which in turn triggers the up-regulation of *tapA* resulting in the production of matrix. Importantly, there are strong trade-offs between *srfA* and *tapA*, which likely underpins why cells segregate tasks in this system (van Gestel et al., 2015b).

In a more recent study using *Pseudomonas fluorescens*, similar cooperative interactions evolved that affected migration and led to increased colony-wide mobility and fitness (Kim et al., 2014, 2016). Notably, the cooperative division of labor that was seen in these experiments arose *de novo* during laboratory evolution, indicating that divisions of labor can appear stably with relative ease over very short evolutionary timeframes. Here, rather than surfactin and matrix production, D-cells, corresponding to a “dry” morphotype, evolved from an otherwise homogeneous parental M-cell that expressed a mucoid morphology. While D-cells grew within the center of the colony, forming a fan-like structure, M-cells were pushed toward the colony edge. This lead to increased mobility of the entire colony, greater acquisition of territory, and presumably increased access to resources at the colony edge. Amazingly, the evolution of this division of labor required only a single nucleotide mutation for D-cells to arise, which altered the concentrations of the intracellular messenger cyclic-di-GMP and thereby potentially changed the expression of hundreds of genes directly or indirectly regulated by this second messenger. This result underscores the fact that although divisions of labor can result in highly complex and tightly orchestrated phenotypes, they can arise by very few mutational events, although these mutations may be accompanied by highly pleiotropic effects (Kim et al., 2016). In addition, we anticipate that this type of experimental study will be instrumental in helping to identify the ecological conditions that facilitate the emergence of microbial divisions of labor.

## Conclusions and Perspective

Microbial divisions of labor have been best studied in the more general context of microbial sociality and multicellularity. The diverse examples presented above and shown in **Supplementary Table 1** from different systems and functional roles highlight that divisions of labor rely on high relatedness and kin selection. However, although kin selection provides a powerful explanation for how divisions of labor are maintained, this approach is less able to explain the extensive variation observed in divisions of labor across different genotypes within species. Equally, it is limited in its ability to explain the conditions under which divisions of labor arise. ESS (evolutionarily stable strategy) models may help to resolve these questions (West and Cooper, 2016), an effort that must be further informed by a greater understanding of the ecological benefits of divisions of labor in the conditions where they evolved. Finally, given the apparent ease with which divisions of labor evolve in the laboratory (Kim et al., 2016), experimental evolution offers unparalleled opportunities to address these questions mechanistically and phenotypically in highly tractable experimental systems.

## Author Contributions

All authors listed, have made substantial, direct and intellectual contribution to the work, and approved it for publication.

## Conflict of Interest Statement

The authors declare that the research was conducted in the absence of any commercial or financial relationships that could be construed as a potential conflict of interest.
