# Supplementary material for: Understanding Microbial Divisions of Labor
Source: Front Microbiol. 2016 Dec 21;7:2070. doi: 10.3389/fmicb.2016.02070 (PMC5174093; doi:10.3389/fmicb.2016.02070)
Supplement: Supplementary file 1 [file Table_1.DOCX]

| **Supplemental Table 1:** A selection of studies examining microbial divisions of labour across different model systems. Prokaryotic examples are shown above while eukaryotic examples are shown below in tan. | | | | | |
| --- | --- | --- | --- | --- | --- |
| **Species** | **Colony-level benefits** | **Cooperative cell types** | **Interactions between cells** | **References** | **Evidence: Benefits of division of labour** |
| *Bacillus subtilis* | Flagellum-independent migration on solid surfaces | Surfactin-producing cells;  Matrix-producing cells | Surfactin-producing cells lubricate cells and substrate to allow matrix-producing cells to form van Gogh bundles for colony expansion | (van Gestel et al., 2015b) | Experimentally confirmed |
| *Pseudomonas fluorescens* | Mobility and population size | D-cells (Dry);  M-cells (Mucoid) | D-cells push M-cells during colony spreading while M-cells produce a mucoid polymer to reduce resistance to movement | (Kim et al., 2016) | Experimentally confirmed |
| *Anabaena* spp. | Growth under depletion of usable nitrogen | Vegetative cells  Heterocysts | Heterocysts fix nitrogen while vegetative cells fix carbon. These products are shared. | (Flores and Herrero, 2010; Herrero et al., 2016) | Experimentally confirmed in certain conditions. |
| *Salmonella typhimurium* | Cooperative virulence/Antibiotic-tolerance | Virulent and avirulent cells | Host colonization/antibiotic tolerance | (Arnoldini et al., 2014; Stecher et al., 2007) | Experimentally confirmed |
| *Myxococcus xanthus* | Reproduction/Dispersal | Spore cells  Peripheral rod cells  Lysed cells | Stalk cells are assumed to undergo PCD, thereby potentially facilitating dispersal and/or spore survival. | (Shimkets, 1990; Velicer and Vos, 2009) | Widely cited as a clear division of labour, but colony-level benefits remain to be quantified. Possible dispersal benefits. |
| *Pseudomonas aeruginosa* | Biofilm/Microcolony structure | Motile  Non-motile cells | Non-motile cells form a stalk, while motile cells migrate to the top of the microcolony where they form a mushroom-like cap.  Non-motile cells produce quorum-sensing signals, siderophores, surfactants and polysaccharides to support motile cells. | (van Gestel et al., 2015a; Klausen et al., 2003) | Colony-level benefits of phenotypic heterogeneity remain uncertain. Increased individual nutrient access. |
| *Pseudomonas aeruginosa* | Dispersal/Virulence/Iron acquisition | Dispersing cells  Lysed cells | Localized cell lysis and siderophore production facilitates microcolony formation and cell dispersal. | (Sauer et al., 2002) | Colony-level benefits remain to be clarified. Possible dispersal benefits. |
| *Streptomyces* spp. | Nutrient acquisition /Dispersal | Vegetative hyphae  Aerial hyphae  Spores | Vegetative hyphae grow into the substrate and secrete enzymes to break down inorganic nutrients. Under nutrient depletion, these hyphae produce antibiotics and undergo PCD that are believed to provide nutrients for the subsequent growth of aerial hyphae, which differentiate into spore chains. | (Barka et al., 2016; Claessen et al., 2014) | Colony-level benefits remain to be clarified. Mechanisms of PCD unclear. |
| *Aspergillus* spp. | Nutrient acquisition /Dispersal | Vegetative hyphae  Aerial hyphae  Conidia | Vegetative hyphae absorb nutrients from the environment and grow into the air as aerial hyphae which sporulate as conidia for dispersal. | (Bennett, 2010; Krijgsheld et al., 2013) | Colony-level benefits remain to be quantified. |
| *Cryptococcus gattii* | Pathogenicity/Virulence | Tubular mitochondrial morphology;  Non-tubular mitochondrial morphology | A sub-population of cells undergoes mitochondrial tubularisation which enhances the proliferation of remaining cells within macrophages. | (Voelz et al., 2014) | Mechanisms confirmed, but the population benefits of increased virulence are uncertain. |
| *Dictyostelium discoideum* | Reproduction/Dispersal | Stalk cells  Spore cells | Stalk cells undergo PCD and elevate spores above the substrate. | (Strassmann, 2016) | Widely cited as a clear division of labour, but colony-level benefits remain to be confirmed. Possible dispersal benefits. |

**References**

Arnoldini, M., Vizcarra, I. A., Peña-Miller, R., Stocker, N., Diard, M., Vogel, V., et al. (2014). Bistable Expression of Virulence Genes in *Salmonella* Leads to the Formation of an Antibiotic-Tolerant Subpopulation. *PLoS Biol.* 12, e1001928. doi:10.1371/journal.pbio.1001928.

Barka, E. A., Vatsa, P., Sanchez, L., Gaveau-Vaillant, N., Jacquard, C., Klenk, H.-P., et al. (2016). Taxonomy, Physiology, and Natural Products of Actinobacteria. *Microbiol. Mol. Biol. Rev.* 80, 1–43. doi:10.1128/MMBR.00019-15.

Bennett, W. J. (2010). An Overview of the Genus Aspergillus. *Aspergillus Mol. Biol. Genomics*, 1–17. doi:doi:10.1201/9781420008517.sec1.

Claessen, D., Rozen, D. E., Kuipers, O. P., Søgaard-Andersen, L., and van Wezel, G. P. (2014). Bacterial solutions to multicellularity: a tale of biofilms, filaments and fruiting bodies. *Nat. Rev. Microbiol.* 12, 115–124. doi:10.1038/nrmicro3178.

Flores, E., and Herrero, A. (2010). Compartmentalized function through cell differentiation in filamentous cyanobacteria. *Nat. Rev. Microbiol.* 8, 39–50. doi:10.1038/nrmicro2242.

van Gestel, J., Vlamakis, H., and Kolter, R. (2015a). Division of Labor in Biofilms: the Ecology of Cell Differentiation. *Microbiol. Spectr.* 3, MB-0002-2014. doi:10.1128/microbiolspec.MB-0002-2014.

van Gestel, J., Vlamakis, H., Kolter, R., Couzin, I., Nadell, C., Bucci, V., et al. (2015b). From Cell Differentiation to Cell Collectives: *Bacillus subtilis* Uses Division of Labor to Migrate. *PLOS Biol.* 13, e1002141. doi:10.1371/journal.pbio.1002141.

Herrero, A., Stavans, J., and Flores, E. (2016). The multicellular nature of filamentous heterocyst-forming cyanobacteria. *FEMS Microbiol. Rev.* 40.

Kim, W., Levy, S. B., and Foster, K. R. (2016). Rapid radiation in bacteria leads to a division of labour. *Nat. Commun.* 354, 1395–1405. doi:10.1038/ncomms10508.

Klausen, M., Aaes-Jørgensen, A., Molin, S., and Tolker-Nielsen, T. (2003). Involvement of bacterial migration in the development of complex multicellular structures in *Pseudomonas aeruginosa* biofilms. *Mol. Microbiol.* 50, 61–8. Available at: http://www.ncbi.nlm.nih.gov/pubmed/14507363 [Accessed November 16, 2016].

Krijgsheld, P., Bleichrodt, R., van Veluw, G. J., Wang, F., Müller, W. H., Dijksterhuis, J., et al. (2013). Development in *Aspergillus*. *Stud. Mycol.* 74, 1–29. doi:10.3114/sim0006.

Sauer, K., Camper, A. K., Ehrlich, G. D., Costerton, J. W., and Davies, D. G. (2002). *Pseudomonas aeruginosa* Displays Multiple Phenotypes during Development as a Biofilm. *J. Bacteriol.* 184, 1140–1154. doi:10.1128/jb.184.4.1140-1154.2002.

Shimkets, L. J. (1990). Social and developmental biology of the myxobacteria. *Microbiol. Rev.* 54, 473–501. Available at: http://www.ncbi.nlm.nih.gov/pubmed/1708086 [Accessed October 6, 2016].

Stecher, B., Robbiani, R., Walker, A. W., Westendorf, A. M., Barthel, M., Kremer, M., et al. (2007). *Salmonella enterica* Serovar Typhimurium Exploits Inflammation to Compete with the Intestinal Microbiota. *PLoS Biol.* 5, e244. doi:10.1371/journal.pbio.0050244.

Strassmann, J. E. (2016). Kin Discrimination in *Dictyostelium* Social Amoebae. *J. Eukaryot. Microbiol.* 63, 378–383. doi:10.1111/jeu.12307.

Velicer, G. J., and Vos, M. (2009). Sociobiology of the Myxobacteria. *Annu. Rev. Microbiol.* 63, 599–623. doi:10.1146/annurev.micro.091208.073158.

Voelz, K., Johnston, S. A., Smith, L. M., Hall, R. A., Idnurm, A., and May, R. C. (2014). “Division of labour” in response to host oxidative burst drives a fatal *Cryptococcus gattii* outbreak. *Nat. Commun.* 5, 5194. doi:10.1038/ncomms6194.
